# The role of ferroptosis in neurodegenerative diseases

**DOI:** 10.3389/fncel.2024.1475934

**Published:** 2024-10-15

**Authors:** Yifan Fei, Yifei Ding

**Affiliations:** School of Life Sciences, Zhejiang Chinese Medical University, Hangzhou, China

**Keywords:** ferroptosis, regulated cell death, ferroptosis inhibitor, inhibitor, neurodegenerative diseases

## Abstract

Ferroptosis represents an iron^−^ and lipid peroxidation (LPO)-mediated form of regulated cell death (RCD). Recent evidence strongly suggests the involvement of ferroptosis in various neurodegenerative diseases (NDs), particularly Alzheimer’s disease (AD), Parkinson’s disease (PD), Huntington’s disease (HD), multiple sclerosis (MS), and amyotrophic lateral sclerosis (ALS), among others. The treatment of ferroptosis poses both opportunities and challenges in the context of ND. This review provides a comprehensive overview of characteristic features, induction and inhibition of ferroptosis, highlighting the ferroptosis inhibitor and the underlying mechanisms responsible for its occurrence. Moreover, the review explores how these mechanisms contribute to the pathogenesis and progression of major neurodegenerative disorders. Additionally, it presents novel insights into the role of ferroptosis in ND and summarizes recent advancements in the development of therapeutic approaches targeting ferroptosis. These insights and advancements hold potential to guide future strategies aimed at effectively managing these debilitating medical conditions.

## 1 Introduction

Cells serve as the fundamental building blocks of life, embodying paramount significance in every facet thereof, owing to their indispensable roles in proliferation, differentiation, functional attributes, and ultimately cell death. Cells possess the inherent capability to experience two distinct modes of cell death: accidental cell death (ACD) and RCD (Tang et al., 2019; Mishra et al., 2018; Bedoui et al., 2020). ACD represents an uncontrolled and unrestrained biological process, whereas RCD encompasses meticulously orchestrated signaling cascades and well-defined effector mechanisms operating at the molecular level (Tang et al., 2019; Mishra et al., 2018; Bedoui et al., 2020). Recent scientific investigations have elucidated a burgeoning repertoire of groundbreaking non-apoptotic manifestations of RCD, garnering increasing recognition for their involvement in a myriad of human diseases. Among these RCD variants, apoptosis is the most extensively studied one, characterized by a reduction in cellular volume, chromatin condensation, and the emergence of apoptotic bodies (Xie et al., 2016a; Abou-Ghali and Stiban, 2015). Nevertheless, numerous alternative non-apoptotic modalities of RCD are currently under investigation, and among them, ferroptosis has emerged as a prominent research focus, which is an iron-dependent mechanism of cellular demise that executes independently of apoptosis (Bedoui et al., 2020).

Ferroptosis was first discovered as the mechanism by which erastin and RAS-selective lethal 3 (RSL3), synthetic lethal compounds, selectively kill oncogenic rat sarcoma (RAS) mutant cells (Dixon et al., 2012). It is characterized by its smaller mitochondria, reduced condition, increased membrane density, and mitochondrial membrane rupture. The differences between apoptosis and ferroptosis are shown in Table 1. However, the nucleus remains unaffected in ferroptosis. Ferroptosis can occur in various ways and under different conditions, including both physiological and pathological states. The physiological processes affected by ferroptosis can be categorized as follows: 1. Promotion of anti-viral immunity through selenium supplementation: When Cluster of Differentiation 4^+^ (CD4^+^) TFH cells are exposed to a selenium-rich diet, the expression of selenoprotein glutathione peroxidase 4 (GPX4) increases (Stockwell, 2022). This protein helps to suppress ferroptosis, leading to an increase in memory B cells and long-lasting viral immunity (Stockwell, 2022). 2. Role of ferroptosis in tumor suppression: A diet rich in polyunsaturated fatty acids (PUFA) promotes the production of polyunsaturated fatty acids-containing phospholipids (PUFA-PLs) (Stockwell, 2022). These compounds facilitate tumor ferroptosis. Additionally, Cluster of Differentiation 8^+^ (CD8^+^) T cells contribute to tumor cell ferroptosis by releasing Interferon-*γ* (IFNγ) and AA (Stockwell, 2022). 3. Ferroptosis associated with aging: As rats and mice age, they experience an increase in ferroptosis markers (Stockwell, 2022). 4. Involvement of ferroptosis in development: Nucleated erythrocytes undergo ferroptosis before enucleation and erythrocyte maturation (Stockwell, 2022). Ferroptosis that occurs under abnormal conditions often leads to diseases such as Sedaghatian-type spondylometaphyseal dysplasia (SSMD) and Systemic lupus erythematosus (SLE) (Stockwell, 2022). Furthermore, there have been numerous studies on ferroptosis in tumors. Inducing iron death can lead to the therapeutic death of tumor cells. For example, a diffuse large B cell lymphoma xenograft was sensitive to treatment with the system xc^−^ inhibitor imidazole ketone erastin (IKE), which activated markers of ferroptosis in the xenografted tumors (Stockwell, 2022; Zhang et al., 2019; Badgley et al., 2020). Knockout of solute carrier family 7 member 11 (SLC7A11) provided significant benefit in a mouse genetic model of pancreatic cancer, without inducing markers of other types of cell death beyond ferroptosis (Stockwell, 2022; Zhang et al., 2019; Badgley et al., 2020). In addition to tumors, ferroptosis is also implicated in a variety of ND. Iron chelators and lipid-soluble antioxidants have been effective in suppressing ferroptosis, highlighting the involvement of iron dyshomeostasis and lipid peroxide accumulation in this process. In conclusion, the suppression of ferroptosis holds potential in preventing the initiation and progression of diseases.

**Table 1:** The differences between apoptosis and ferroptosis.

	Morphological features	Key biochemical pathway components
Apoptosis	Nuclear rupture, plasma membrane blistering, cell shrinkage, apoptotic body formation and phagocytosis of neighboring cells.	Proapoptotic B-cell lymphoma-2 (BCL-2) family members, caspase activation, cleavage of hundreds of caspase substrates (e. g. intracranial atherosclerotic disease (ICAD), Poly ADP-ribose polymerase (PARP)), phosphatidylserine exposure, Mitochondrial Membrane Potential (△ψm) dissipation, Manganese-Based Tumor Immunotherapy (MOMP) and ROS production.
Ferroptosis	Smaller mitochondria, reduced condition, increased membrane density, mitochondrial membrane rupture, but normal nucleus.	Iron accumulation, cysteine deprivation, and/or glutathione peroxidase inactivation eventually lead to lipid peroxidation.

ND is one of the leading causes of death in modern societies (Varela and Garcia-Rendueles, 2022), and has become an important public health burden, due to its increasing incidence and mortality and an associated rise in healthcare costs (GBD 2016 Neurology Collaborators, 2019; Yang et al., 2020). Therefore, it is urgent to seek an exact pathogenesis and treatment. ND is characterized by the gradual decline of motor and cognitive functions, resulting from the degeneration and loss of neurons in the central nervous system (CNS) (Bianchi et al., 2021). AD, PD, HD, MS and ALS are the most common ND (Wang et al., 2023a). Several notable features of ND, such as LPO and iron dyshomeostasis, align with the characteristics of ferroptosis, suggesting a potential involvement of ferroptosis in the progression of these diseases. Aging constitutes the primary risk factor for neurodegenerative ailments and is accompanied by cerebral iron accumulation (Buijs et al., 2017). Likewise, different ND have reported instances of iron accumulation in affected brain regions (Chen et al., 2024; Mohan et al., 2024; Rogers et al., 2019; Masaldan et al., 2019). Ferroptosis inhibitors have demonstrated their protective efficacy in cellular or animal AD, PD, and HD models (Skouta et al., 2014; Ding et al., 2023; Reichert et al., 2020). Hence, it is plausible that ferroptosis assumes a pivotal function in the development of diverse neurodegenerative disorders.

Herein, the main objective of this paper is to offer a comprehensive understanding of ferroptosis and its significance in ND. Additionally, it also aims to present some new insights into ferroptosis in ND.

## 2 Ferroptosis

### 2.1 The features of ferroptosis

Ferroptosis is an exclusive form of RCD that is distinguished by both morphological and mechanistic differences when compared to apoptosis and other types of RCD. In terms of morphology, cells undergoing ferroptosis do not exhibit the customary apoptotic characteristics such as chromatin condensation and apoptotic body formation. Instead, they are marked by contracted mitochondria and a reduction in the quantity of mitochondrial cristae. The lethal accumulation of lipid peroxides serves as a pivotal trait of ferroptosis and revolves around a competition between cellular activities that promote ferroptosis and the antioxidant-buffering capabilities offered by ferroptosis defense systems (Punziano et al., 2024). Ferroptosis occurs when the promoting activities of ferroptosis surpass the ability of defense systems to alleviate oxidative stress. It is this mechanism that distinguishes ferroptosis from other forms of RCD, which usually involve the initiation of specific cell death executioner proteins. Prominent examples of these proteins include caspases in apoptosis, mixed lineage kinase domain-like protein (MLKL) in necroptosis, and gasdermin D in pyroptosis (Tonnus et al., 2019; Aachoui et al., 2013; Evavold et al., 2018). Furthermore, cells undergoing ferroptosis exhibit distinctive profiles of oxidized phospholipids (PL), setting them apart from cells undergoing other types of cell death (Tonnus et al., 2019; Aachoui et al., 2013; Evavold et al., 2018). This further underscores the exceptional nature of ferroptosis as a RCD pathway.

The search for physiological markers associated with ferroptosis is essential. Various markers can be utilized, including LPO markers such as Thiobarbituric acid reactive substances (TBARS), C11-BODIPY, Liperfluo fluorescence, Liquid Chromatography-Mass Spectrometry (LC–MS)/MS lipidomics, Anti-melanoma differentiation-associated protein (anti-MDA) adduct antibody staining (Kobayashi et al., 2021; Yamada et al., 2001), and anti-4 Hydroxynonenal (anti-HNE) adduct antibody staining (Zheng et al., 2021). Additionally, markers of mitochondria, like Shrunken, dense mitochondria, and markers of TfR1 can be identified through anti-TfR1 antibody staining (Feng et al., 2020). During ferroptosis, several genes are induced, including ChaC glutathione specific gamma-glutamylcyclotransferase 1 (CHAC1), prostaglandin-endoperoxide synthase 2 (PTGS2), SLC7A11, and acyl-CoA synthetase long-chain family member 4 (ACSL4), while Regulator of G protein signaling 4 (RGS4) is downregulated. The altered expression of these genes can be detected using Realtime fluorescence quantitative PCR (qPCR), providing indicators of ferroptosis (Stockwell, 2022; Dou et al., 2022). Another article reports that over-oxidized stimulates iron death by inhibiting cystine uptake, which makes Peroxiredoxin-3 (PRDX3) peroxide also a specific marker for ferroptosis (Cui et al., 2023).

### 2.2 The induction of ferroptosis

**Figure 1:**
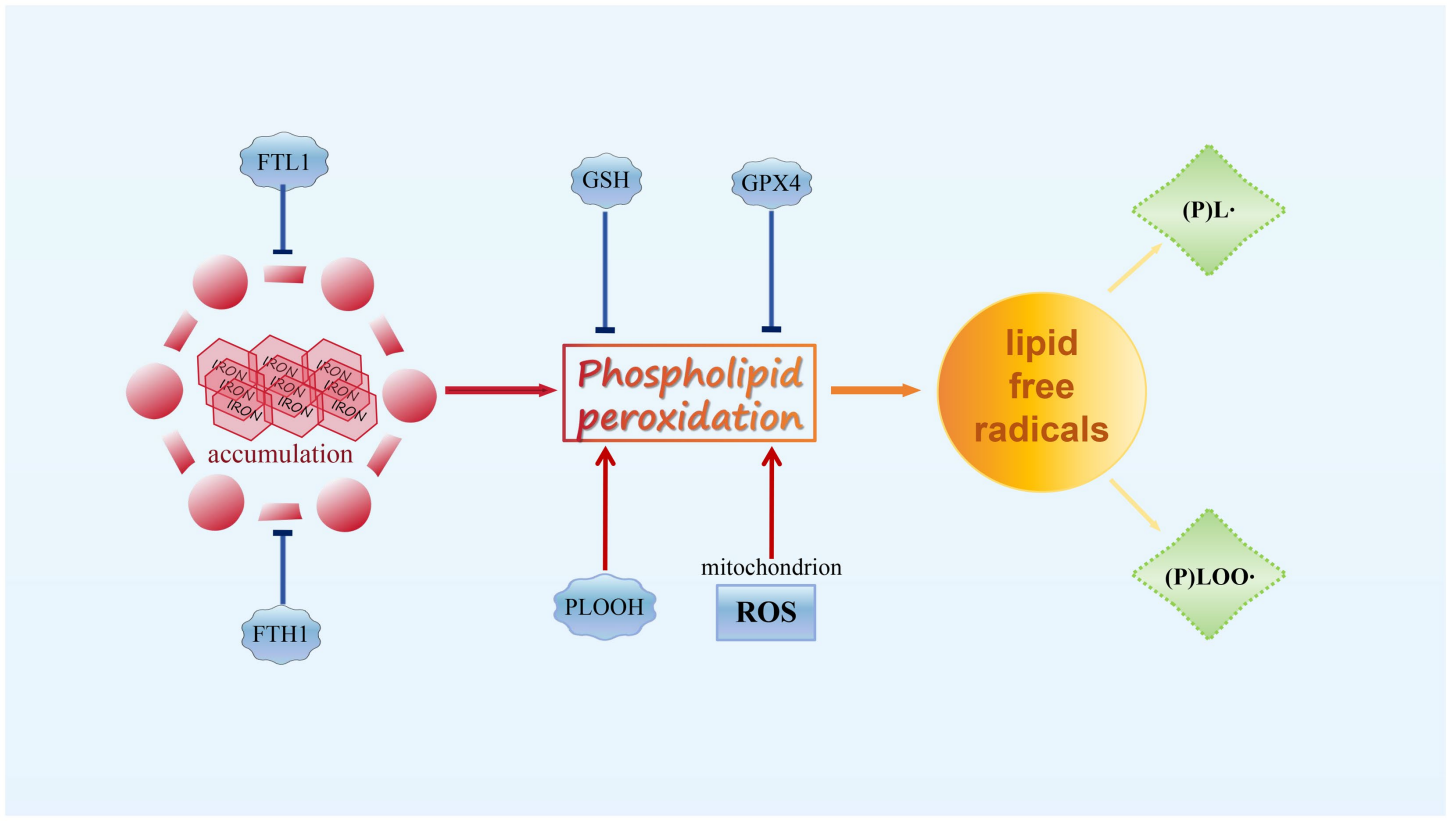
The principle of ferroptosis.

Ferroptosis is a cellular death mechanism that relies on the iron-induced oxidation of lipids (Stockwell, 2022; Yang et al., 2016; Kim et al., 2023), and the principle of ferroptosis is showed in Figure 1. The central process in ferroptosis execution is the iron-catalyzed peroxidation of PL that contain PUFA (Stockwell, 2022; Yang et al., 2016; Kim et al., 2023).

Under physiological conditions, lipids undergo constant peroxidation through non-enzymatic autoxidation or lipoxygenase (LOX)-mediated LPO, especially during cellular stress. However, the potentially harmful lipid hydroperoxides produced are continually monitored by the selenoenzyme GPX4. This unique enzyme directly reduces lipid hydroperoxides in the acyl chains of PL to lipid alcohols while oxidizing reduced glutathione (GSH) to form glutathione disulfide (Kim et al., 2023; Anthonymuthu et al., 2021). However, when GPX4 is deactivated or GSH levels are depleted in cells, phospholipid peroxides (PLOOH) accumulate, leading to the production of lipid radicals, such as lipid peroxyl radicals ((P)LOO∙) and alkyl radicals ((P)L∙), through iron-dependent free radical chain reactions. Uncontrolled LPO and the generation of reactive aldehydes due to LPO ultimately lead to cell membrane rupture and cell death through ferroptosis (Conrad and Pratt, 2019; Chu et al., 2019). Although lipid radicals are the primary contributors to ferroptosis, their origins are not yet well understood. Mitochondria are believed to be the main generators of reactive oxygen species (ROS) in a cell due to incomplete reduction of molecular oxygen (Shah et al., 2018; Zou et al., 2020; Yan et al., 2021). Therefore, mitochondrial ROS is essential for ferroptosis. Notably, blocking ferroptosis induced by GSH depletion can be achieved by inhibiting the electron transport chain, which releases electrons to generate superoxide anion (O2^−^∙) (Gan, 2021; Gao et al., 2019). Superoxide is primarily localized in the mitochondrial matrix and generated in the intermembrane space through complex III. It can then be transported from mitochondria to the cytoplasm through voltage-dependent anion channels (Schaar et al., 2015; Dan Dunn et al., 2015). However, the precise mechanisms by which mitochondrial superoxide is released into the cytoplasm or at a membrane to induce LPO are still unknown. Besides mitochondrial ROS, the rate of ferroptosis is also influenced by glutaminolysis and the tricarboxylic cycle (Gao et al., 2024). Disruption of oxidative phosphorylation by deleting cytochrome c oxidase assembly factor 10 leads to lysosomal and mitochondrial defects, resulting in LPO and ferroptosis of cardiac cells (Ahola et al., 2022). There are numerous pathways through which ferroptosis can be stimulated, involving diverse mechanisms.

Four main mechanisms have been identified for the induction of ferroptosis, including hindering system xc^−^, inhibiting, degrading, or inactivating GPX4, depleting reduced Coenzyme Q10 (CoQ10), and triggering LPO via peroxides, iron, or PUFA overload (Stockwell, 2022). There is ample evidence supporting that inhibiting system xc^−^ is a potent mechanism for triggering ferroptosis. Various small molecule inhibitors of this antiporter, such as erastin, sulfasalazine, and glutamate, effectively block system xc^−^ and thereby induce ferroptosis. Moreover, cystine depletion eliminates the extracellular substrate for system xc^−^ and also promotes ferroptosis. Similarly, the genetic inactivation or small-molecule-mediated inhibition or degradation of GPX4 leads to ferroptosis in multiple cell types. In cases where GPX4 is absent, the inhibition of CoQ10 biosynthesis through the mevalonate pathway or the inactivation of CoQ10 reductases, such as Ferroptosis suppressor protein 1 (AIFM2/FSP1) or Dihydroorotate Dehydrogenase (DHODH), initiates ferroptosis. Finally, an excess of iron, PUFAs, or peroxides, such as Tert-butyl hydroperoxide (tBOOH) or FINO2, induces ferroptosis (Shah et al., 2018). All four of these mechanisms exhibit significant specificity in inducing ferroptosis compared to other forms of cell death. This specificity is evident as the lethal effects of these mechanisms are mostly or entirely neutralized by ferroptosis-specific inhibitors, and markers of alternative cell death mechanisms are not activated.

### 2.3 The inhibition of ferroptosis

Biological processes exert control over ferroptosis by modulating the molecules involved in its promotion or surveillance, as well as redox and iron homeostasis, and cell metabolism. It is widely acknowledged that numerous signaling pathways dictate a cell’s susceptibility to ferroptosis in specific biological circumstances, supported by an ever-expanding body of evidence. According to the principle of ferroptosis, the inhibitors can be classified into three types: first, drugs that prevent iron accumulation at the source of ferroptosis; second, drugs that affect LPO by directly inhibiting ROS accumulation or indirectly inhibiting factors that influence the body, like enhancing the GPX4/GSH axis; and third, drugs that directly eliminate lipid free radicals to inhibit ferroptosis (Table 2) (Liang et al., 2023).

**Table 2:** Ferroptosis inhibitors.

Compound/drug	Proposed mechanisms of ferroptosis inhibition	Model system in which ferroptosis inhibition has been proven		References
		*In Vitro*	*In Vivo*	
All-trans-retinoic acid	Decreased lipid peroxidation	Mouse hippocampal cell line (HT-22) and human glioblastoma (U-251)	–	Jakaria et al. (2023)
Aloe-emodin	Decreased lipid peroxidation, and increased expression of GPX4 and Nrf2	Rat cardiomyocytes (PC-12)	–	He et al. (2023)
Alpha-tocopherol	Improved the ROS accumulation, iron overload, lipid peroxidation and mitochondrial dysfunction	Rat cardiomyocytes (PC-12)	–	Zhu et al. (2024)
Artepillin C	Decreased ROS production	Mouse hippocampal neuronal cell line (HT-22)	–	Takashima et al. (2019)
Astragaloside-IV	Decreased lipid peroxidation, ROS production, GSH depletion, increased expression of Nrf2, and maintained normal mitochondria structure	Retinal segment endothelial cell line (ARPE-19)	–	Tang et al. (2022)
Astringin	Decreased ROS accumulation	Rat bone marrow-derived mesenchymal stem cells bmMSCs	–	Chen et al. (2021b)
Baicalein	Decreased lipid peroxidation, iron accumulation, and GSH depletion, and GPX4 degradation	Human pancreatic adenocarcinoma cell lines (PANC1; BxPc3)	–	Xie et al. (2016b)
	Decreased phosphatidylethanolamine oxidation	–	Mice were treated with baicalein via intraperitoneal injection after ferroptosis was induced by traumatic brain injury. Ferroptosis was examined in pericontusional cortex tissue.	Kenny et al. (2019)
Baicalin	Decreased lipid peroxidation, ROS, and iron accumulation, and increased expression of GPX4	Rat cardiac myoblast cells (H9c2)	Rats were treated with baicalin orally before myocardial ischemia/reperfusion injury was induced. Ferroptosis markers were evaluated in heart tissue.	Fan et al. (2021)
Beta-caryophyllene	Decreased iron accumulation, GSH depletion, and ROS production, upregulated expression of GPX4 and Nrf2, and maintained normal mitochondria structure	Primary astrocytes which were derived from postnatal SD rats	Mice were treated with beta-caryophyllene intragastrically after ferroptosis was induced by cerebral ischemia reperfusion. Ferroptosis was examined in brain tissues.	Hu et al. (2022)
BRD4770	Decreased lipid peroxidation and upregulated GPX4 and FSP1 mRNA	–	Mice were treated with BRD4770 via intraperitoneal injection after ferroptosis was induced by aortic dissection. Ferroptosis markers were evaluated in aortae tissue.	Chen Y. et al. (2022)
Butein	Inhibition of lipid peroxidation	Rat bone marrow-derived mesenchymal stem cells	–	Liu J. et al. (2020)
Butylated hydroxytoluene	Prevented oxidation of membrane lipids	Human neuroblastoma cell line (SH-SY5Y)	–	Faraji et al. (2024)
	No specific study (but found butylated hydroxytoluene normalized the expression of ferroptosis-related genes in a Rat Alzheimer’s disease model)	–	Mice were treated with butylated hydroxytoluene orally before ferroptosis was induced by Alzheimer’s disease. Ferroptosis markers were evaluated in hippocampus.	Faraji et al. (2024)
Cardamonin	Decreased GSH depletion, iron accumulation, and ROS production, upregulated expression of GPX4, and maintained normal mitochondria structure	Chondrocytes which were obtained from five-days-old Sprague–Dawley (SD) rats	Rats were treated with cardamonin by intra-articular injection after heart failure was induced by osteoarthritis. Ferroptosis markers were evaluated in knee joint.	Gong et al. (2023)
Carnosic acid	Decreased GSH depletion, lipid peroxidation, iron accumulation, and ROS production, and upregulated expression of Nrf2	Rat adrenal gland irregularly shaped cell line (PC-12)	–	Cheng et al. (2021)
Carthamin	Decreased lipid peroxidation, ROS production, GSH depletion, and iron accumulation, and increased expression of GPX4	–	Rats were treated with carthamin orally before cerebral ischemia–reperfusion injury was induced. Ferroptosis markers were evaluated in brain tissue.	Guo et al. (2021)
Chebulagic and chebulinic acids	Decreased ROS accumulation	Rat bone marrow-derived mesenchymal stem cells bmMSCs	–	Yang et al. (2021)
Chrysophanol	Decreased iron accumulation and ROS production, and increased expression of GPX4	Human renal proximal tubular epithelial cell line (HK-2 cells)	–	Lin et al. (2021)
Curcumin	Decreased iron accumulation, lipid peroxidation, and GSH depletion, and increased expression of GPX4	Mouse pancreatic β-cell line (MIN6)	–	Kose et al. (2019)
	Decreased lipid peroxidation and GSH depletion	–	Mice were treated with curcumin via intraperitoneal injection after ferroptosis was induced by rhabdomyolysis. Ferroptosis markers were evaluated in kidney tissue.	Guerrero-Hue et al. (2019)
Cyanidin-3-glucoside	Decreased ROS production, lipid peroxidation, and iron accumulation, and increased expression of GPX4	Rat cardiac myoblast cells (H9c2)	Rats were treated with cyanidin-3-glucoside via intraperitoneal injection before myocardial ischemia–reperfusion injury was induced. Ferroptosis markers were evaluated in heart tissue.	Shan et al. (2021)
Deferiprone	Decreased iron accumulation	–	Mice were treated with deferiprone via intraperitoneal injection after ferroptosis was induced by demyelination. Ferroptosis markers were evaluated in optic nerve and eyeball.	Rayatpour et al. (2022)
Deferoxamine	Decreased iron accumulation and GSH depletion, increased expression of GPX4 and xCT	–	Mice were treated with deferoxamine via intraperitoneal injection before ferroptosis was induced by spinal cord injury. Ferroptosis markers were evaluated in spinal cord.	Yao et al. (2019)
	Decreased iron accumulation and improved the structural changes in mitochondria	Mouse chondrocytes	–	Guo et al. (2022)
Dexmedetomidine	Decreased GSH depletion, and upregulated expression of GPX4 and Nrf2	–	Mice were treated with dexmedetomidine by intraperitoneal injection before ferroptosis was induced by sepsis. Ferroptosis was examined in heart tissues.	Wang C. et al. (2020)
	Decrease iron accumulation, ROS production, and GSH depletion, upregulated expression of GPX4 and Nrf2	Rat cardiac myoblast cells (H9c2)	–	Wang Z. et al. (2022)
Ebselen	Decreased lipid peroxidation, iron accumulation, and ROS production, and maintained normal mitochondria structure	Bone-marrow-derived mesenchymal stem cells	–	Huang et al. (2023)
Edaravone	Decreased lipid peroxidation and ROS production	Rat cerebral cortex cells (PC-12)	Mice were treated with edaravone via intraperitoneal injection before ferroptosis was induced by traumatic brain injury. Ferroptosis markers were evaluated in the injured cortex on the striking side.	Shi et al. (2024)
Elabela	Decreased lipid peroxidation and ROS production, increased expression of GPX4 and Nrf2	Cardiac microvascular endothelial cells	Mice were treated with elabela by intraperitoneal injection after ferroptosis was induced by hypertension. Ferroptosis was examined in heart tissues.	Zhang et al. (2022)
Epigallocatechin-3-gallate	Decreased iron accumulation, ROS production, lipid peroxidation, and GSH depletion, and increased expression of GPX4	Rat cardiac myoblast cells (H9c2)	–	He et al. (2021)
	Increased expression of GPX4, maintained normal mitochondria structure, decreased lipid peroxidation, and GSH depletion	–	Mice were treated with epigallocatechin-3-gallate orally before ferroptosis was induced with doxorubicin. Ferroptosis markers were evaluated in heart tissue.	He et al. (2021)
Ferrostatin-1	Increased SLC7A11 and GPX4 expression	Human bronchial epithelial cell line (BEAS-2B)	Mice were treated with ferrostatin-1 via tail vein injection after ferroptosis was induced by acute lung injury. Ferroptosis was examined in lung tissues.	Liu P. et al. (2020)
Fraxetin	Decreased GSH depletion, iron accumulation, and lipid peroxidation, and upregulated GPX4 and Nrf2	Rat cardiac myoblast cells (H9c2)	Rats were treated with fraxetin via intraperitoneal injection before myocardial infarction was induced. Ferroptosis markers were evaluated in heart tissue.	Xu Y. et al. (2021)
Galangin	Decreased lipid peroxidation, GSH depletion, and iron accumulation, and increased expression of GPX4	–	Gerbils were treated with galangin orally after cerebral ischemia–reperfusion injury was induced. Ferroptosis markers were evaluated in hippocampal coronal tissue.	Guan et al. (2021)
Gastrodin	Decreased GSH depletion, lipid peroxidation, iron accumulation, and ROS production, and upregulation of GPX4	Rat glioma cell line (C6)	–	Jiang et al. (2020)
Geraniin	Decrease ROS accumulation, iron accumulation, and lipid peroxidation	Rat bone marrow-derived mesenchymal stem cells bmMSCs	–	Chen et al. (2021a)
Gossypol	Decreased iron accumulation, lipid peroxidation, and ROS production	Rat cardiac myoblast cells (H9c2)	–	Lin et al. (2021)
	Decreased lipid peroxidation and increased expression of GPX4	–	Ferroptosis was induced in rat hearts by ischemia/reperfusion followed by treatment with gossypol acetic acid.	Lin et al. (2021)
Hydrogen sulfide	Decreased GSH depletion, iron accumulation, and ROS production, upregulated expression of GPX4 and Nrf2	Human lung and bronchus epithelial cells (BEAS-2B)	–	Wang Y. et al. (2022)
	Upregulated expression of GPX4 and Nrf2	–	Mice were treated with NaHS by intraperitoneal injection before ferroptosis was induced by PM. Ferroptosis was examined in lung tissues.	Wang Y. et al. (2022)
Icariin	Decreased lipid peroxidation, iron accumulation, and upregulated expression of GPX4 and Nrf2	Human synoviocyte cell line (HUM-CELL-0060)	-	Luo and Zhang (2021)
	Decreased lipid peroxidation, iron accumulation, and ROS production, and upregulated expression of GPX4 and Nrf2	Rat cardiac myoblast cells (H9c2)	–	Liu et al. (2021)
Idebenone	Decreased iron accumulatio and ROS production, increased expression of GPX4 and FSP1	Neonatal rat cardiomyocytes (NRCMs)	Mice were treated with idebenone via oral gavage before ferroptosis was induced by DOX. Ferroptosis markers were evaluated in heart tissue.	Qiu et al. (2024)
Irisin	Decreased ROS production, and increased expression of GPX4 and Nrf2	Human proximal tubular cell line (HK-2)	Mice were treated with irisin intravenously after ferroptosis was induced by acute kidney injury. Ferroptosis was examined in blood and kidney tissues.	Qiongyue et al. (2022)
Isoliquiritigenin	Decreased lipid peroxidation and ROS production, and increased expression of GPX4 and xCT (subunit of xc– transporter), and maintained normal mitochondria structure	Human proximal tubular cell line (HK-2)	–	Tang Y. et al. (2021)
	Decreased lipid peroxidation, ROS production, and iron accumulation and increased expression of GPX4	–	Mice were treated with isoliquiritigenin orally before acute kidney injury was induced. Ferroptosis markers were evaluated in kidney tissue.	Tang Y. et al. (2021)
Kaempferide	Decreased ROS production and increased ARE activation	Mouse hippocampal neuronal cell line (HT-22)	–	Takashima et al. (2019)
Keampferol	Decreased ROS production and increased ARE activation	Mouse hippocampal neuronal cell line (HT-22)	–	Takashima et al. (2019)
	Decreased iron accumulation, lipid peroxidation, and ROS production, and increased expression of GPX4 and Nrf2, and maintained normal mitochondria structure	Primary mouse cortical neurons	–	Yuan et al. (2021)
Liproxstatin-1	Decreased GSH depletion, and increased expression of GPX4	Mouse hippocampal cell line (HT-22)	Mice were treated with liproxstatin-1 via intraperitoneal injection after ferroptosis was induced by subarachnoid hemorrhage. Ferroptosis was examined in brain tissues.	Cao et al. (2021)
Melatonin	Decreased lipid peroxidation, iron accumulation, and attenuated the damage to the mitochondrial structure	Mouse retinal ganglion cell (RGC)	Mice were treated with melatonin via intraocular delivery before ferroptosis was induced by RIR. Ferroptosis markers were evaluated in retina tissue.	Zhang et al. (2023)
Menaquinone-4	Decreased GSH depletion, and increased expression of GPX4	Mice primary neuronal cells	Mice were treated with menaquinone-4 by intraperitoneal injection after ferroptosis was induced by subarachnoid hemorrhage. Ferroptosis was examined in brain tissues.	Zhang et al. (2024)
Morachalcone D	Decreased GSH depletion, ROS production, and iron accumulation, and upregulate GPX4 and NRf2 mRNA	Mouse hippocampal neuronal cell line (HT-22)	–	Wen et al. (2020)
Naringenin	Decreased lipid peroxidation, ROS production, GSH depletion, iron accumulation, and expression of NOX4, and increased expression of GPX4 and Nrf2	–	Rats were treated with naringenin orally before myocardial ischemia–reperfusion injury was induced. Ferroptosis markers were evaluated in myocardial tissue.	Xu S. et al. (2021)
Non-oxidative dopamine	Decreased iron accumulation, lipid peroxidation, and GSH depletion	Human pancreatic cancer cell line (PANC1), human ovarian cancer cell line (HEY) and mouse embryonic fibroblast cell line (MEFs), human embryonic kidney cell line (HEK293 cells) where ferroptosis was induced by erastin	–	Wang et al. (2016)
Nordihydroguaiaretic acid	5-LOX inhibition, decreased lipid peroxidation and ROS production	Human T lymphoblastic leukemic cells (Molt-4; Jurkat)	–	Probst et al. (2017)
Piceatannol	Decreased ROS accumulation	Rat bone marrow-derived mesenchymal stem cells bmMSCs	–	Chen et al. (2021b)
Propofol	Upregulated expression of GPX4 and Nrf2	Rat cardiac myoblast cells (H9c2)	Mice were treated with propofol before ferroptosis was induced by ischemia/reperfusion Injury. Ferroptosis was examined in heart tissues.	Li S. et al. (2022)
Puerarin	Reduce lipid peroxidation, iron accumulation, and expression of NOX4. Increased GPX4 expression	Rat cardiac myoblast cells (H9c2)	Rats were treated with puearin via subcutaneous injection after heart failure was induced by pressure overload. Ferroptosis markers were evaluated in cardiac tissue.	Liu et al. (2018)
Quercetin	Increased GSH levels, decreased lipid peroxidation, and GPX4 depletion	–	Mice were treated with quercetin orally before acute kidney injury was induced. Ferroptosis markers were evaluated in kidney tissue.	Wang Y. et al. (2020)
	Maintained normal mitochondria structure, decreased GSH depletion, lipid peroxidation, and ROS production	Rat kidney epithelial cell line (NRK-52E) and human proximal tubular cell line (HK-2)	–	Wang Y. et al. (2020)
Resveratrol	Decreased lipid peroxidation and iron accumulation, and increased expression of GPX4	Rat cardiac myoblast cells (H9c2)	Rats were treated with resveratrol orally before myocardial ischemia/reperfusion injury was induced. Ferroptosis markers were evaluated in heart tissue.	Li T. et al. (2022)
Salidroside	Decreased iron accumulation, lipid peroxidation, GSH depletion, and ROS production, upregulated expression of GPX4, and maintained normal mitochondria structure	Rat cardiac myoblast cells (H9c2)	Mice were treated with salidroside by intraperitoneal injection before ferroptosis was induced by doxorubicin. Ferroptosis was examined in heart tissues.	Chen H. et al. (2022)
Sterubin	Decreased GSH depletion, ROS accumulation, and free iron concentration	Mouse hippocampal neuronal cell line (HT-22)	–	Fischer et al. (2019)
XJB-5-131	Showed low nanomolar potency against both ferroptosis inducers	Fibrosarcoma cells (HT-1080)	–	Jang et al. (2021)
Zileuton	Inhibited Glutamate-Induced ROS Production, but did not decrease GSH depletion	Mouse hippocampal neuronal cell line (HT-22)	–	Liu et al. (2015)
Zinc	Decreased lipid peroxidation, ROS production and GSH depletion, increased expression of GPX4	Motor neuron cells (VSC4.1)	Mice were treated with ZnG by intraperitoneal injection after ferroptosis was induced by spinal cord injury. Ferroptosis was examined in spinal cord tissues.	Ge et al. (2021)

The main factor affecting iron accumulation is IRP, which is an RNA-binding protein that governs the translation of a cluster of mRNA molecules involved in iron homeostasis by binding to Inositol-requiring enzyme (IRE). Meanwhile, iron regulatory protein (IRP2) acts as the primary detector of labile iron in neurons. In situations where the cellular iron content is insufficient, IRP attaches to IRE located in the 5′ untranslated regions (5′ UTR) of mRNA molecules responsible for iron-responsive proteins, such as Ferritin L chain (FPN), *β*-amyloid precursor protein (APP), and *α*-synuclein, consequently inhibiting the translation of these iron-responsive proteins to reduce iron export and the storage of free iron. Conversely, when intracellular iron levels rise, the IRP and IRE dissociate, leading to an opposing effect characterized by reduced iron absorption and increased free iron storage and export. IRP2 plays an indispensable role in regulating iron within the brain, and its misregulation is closely associated with iron accumulation in ND. In the study of age-related auditory cortical neurodegeneration, it has been discovered that the upregulation of IRP2 augments intracellular iron levels, ultimately leading to ferroptosis (Yao et al., 2021).

Moreover, it has been observed that ferritinophagy promotes ferroptosis. The primary means of intracellular iron storage hinges predominantly on ferritin, which encompasses ferritin light chain polypeptide 1 (FTL1) and ferritin heavy chain polypeptide 1 (FTH1) (Nishizawa et al., 2020). Within the cell, ferritin serves as the premier protein complex for storing iron. Furthermore, iron is released from ferritin primarily through selective autophagy facilitated by nuclear receptor coactivator 4 (NCOA4), a process commonly recognized as ferritinophagy. The complex of ferritin combined with NCOA4 is transported to lysosomes for degradation, subsequently releasing iron for utilization in cellular physiological activities. It has been demonstrated that the intricacy of ferroptosis is affected by NCOA4-mediated ferritinophagy, with studies revealing that the levels of NCOA4 influence the sensitivity to ferroptosis. Suppression of NCOA4 hampers ferritin degradation and curtails ferroptosis, whereas its overexpression heightens the labile iron pool (LIP) (Fang et al., 2021), thereby promoting the accumulation of ROS and the initiation of ferroptosis.

The main ROS related factor is GPX4. Evidence indicates that the activity and stability of GPX4, a vital regulator of ferroptosis, undergo regulation. For instance, persistent oxidative stress and deficiency in glutathione can impair the GSH-dependent reduction of GPX4’s active site selenocysteine, leading to irreversible inactivation through the formation of a redox-dead dehydroalanine, also known as ß-cleavage (Orian et al., 2015). However, the formation of a selenylamide between the selenenic acid and an adjacent amino acid may safeguard the enzyme against irreversible inactivation. Further investigation is required to ascertain if any of these mechanisms are involved in pathological conditions such as ischemia/reperfusion injuries (IRI), although studies have demonstrated that intestinal IRI is associated with reduced GPX4 levels (Li et al., 2019). Additionally, certain substances that induce ferroptosis, including RSL3, can deplete GPX4 through covalent inhibition of its active site selenocysteine, impairment of mevalonate metabolism, or induction of general iron-dependent oxidative stress (Shimada et al., 2016; Gaschler et al., 2018; Chen et al., 2020).

Another significant suppressor of ferroptosis is FSP1, which is regulated by transcription factors Nuclear factor erythroid 2-related factor 2 (NRF2) (Chorley et al., 2012), CCAAT/enhancer-binding protein (CEBP) (Nguyen et al., 2020), and Peroxisome proliferator-activated receptor alpha (PPAR*α*) (Venkatesh et al., 2020). Apart from transcriptional regulation, little is known about the regulation of FSP1’s oxidoreductase activity and how its subcellular localization influences its role in different physiological and pathophysiological processes (Nguyen et al., 2020; Bersuker et al., 2019; Wu et al., 2002; Doll et al., 2019). However, the ability of FSP1 to interact with both reducing and oxidizing substrates (such as Nicotinamide adenine dinucleotide (NADH), nicotinamide adenine dinucleotide phosphate (NADPH), CoQ10, and α-tocopherol) suggests that its regulation is intricate. In particular, it has recently been found that membrane-bound O-acyltransferase domain-containing 1 and 2 (MBOAT1/2) attenuates ferroptosis by bolstering the levels of MUFA present on the cellular membrane (Liang et al., 2023). More importantly, the potential of MBOAT2 to hinder ferroptosis without reliance on GPX4 and FSP1 through a mechanism involving PL remodeling-mediated surveillance has been identified (Liang et al., 2023). Nevertheless, the existing literature on this subject is limited, and additional research is warranted to gain a comprehensive understanding.

## 3 Ferroptosis in ND

ND are a heterogeneous group of complex diseases characterized by neuronal loss and progressive degeneration of different areas of the nervous system. AD, PD, HD, MS, and ALS represent the prevailing neurodegenerative disorders in current medical research. The existence of LPO and iron dyshomeostasis in neurodegenerative disorders exhibits striking parallels to the distinctive traits observed in ferroptosis (Wang et al., 2023a). This suggests that ferroptosis might exert a pivotal role in the advancement of above-mentioned diseases. As might be expected, more recent studies have also looked at the effects of ferroptosis in ND.

### 3.1 The role of ferroptosis in AD

**Figure 2:**
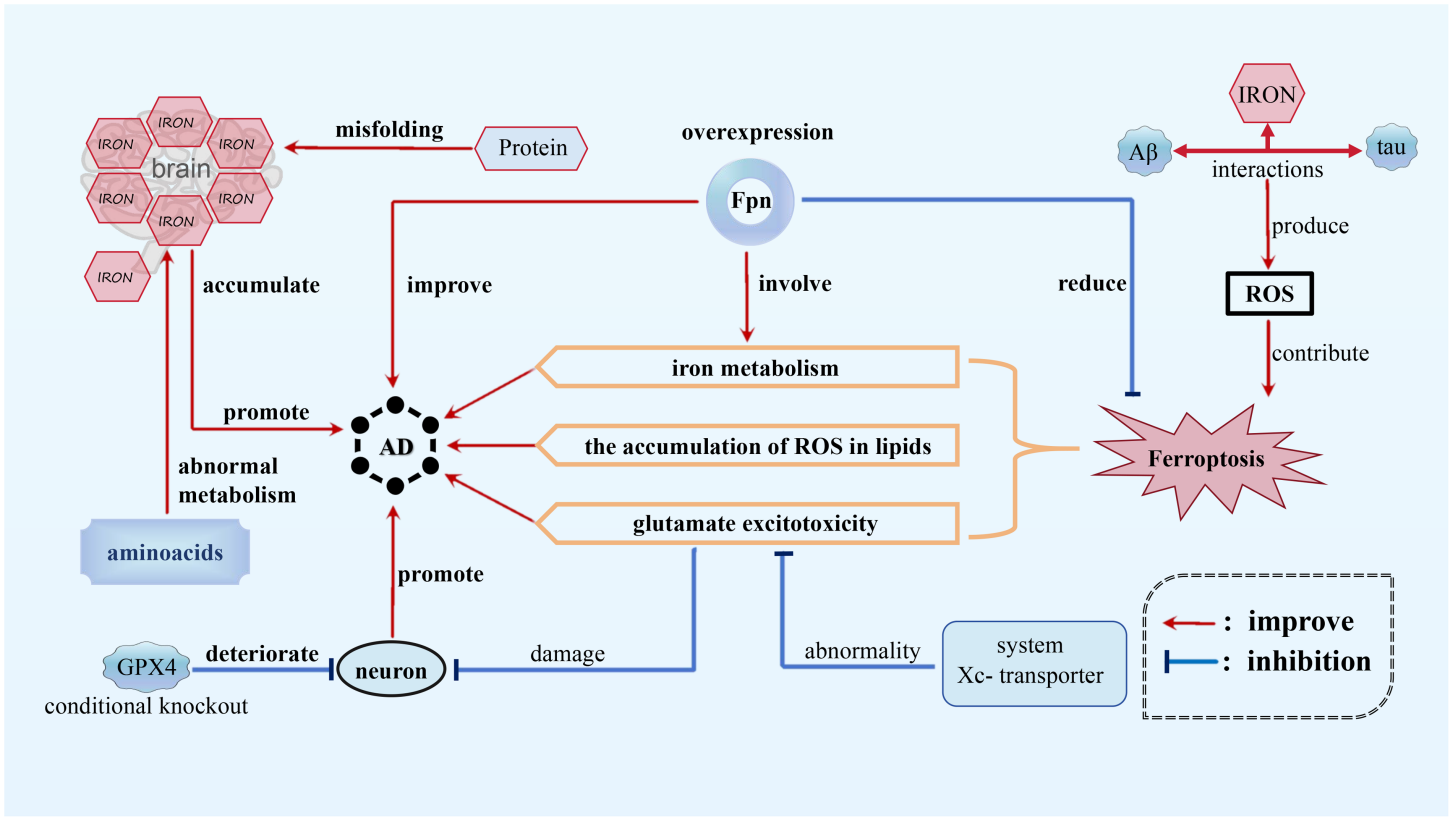
Ferroptosis in AD. In AD, disruptions in the balance of iron within the brain have been observed and higher iron level promotes the progression of AD. Interactions among iron, beta-amyloid (Aβ) protein, and tau protein can provoke ROS, contributing to ferroptosis of neurons. In addition, the abnormality of system Xc-transporter induces glutamate excitotoxicity, which also promotes AD progression. However, FPN overexpression can inhibit ferroptosis, thus alleviating AD.

AD is a chronic, progressive, and irreversible neurodegenerative condition characterized by memory impairment, language disturbances, severe behavioral abnormalities, and deficits in learning abilities (Bao et al., 2021). Ferroptosis, appears to play a significant role in AD by affecting various pathways related to iron metabolism, glutamate excitotoxicity, and the accumulation of ROS in lipids. In the context of AD, disruptions in the balance of iron within the brain have been observed, and higher levels of brain iron have been found to be positively correlated with disease progression and cognitive decline. Interactions among iron, beta-amyloid (Aβ) protein, and tau protein can lead to the production of ROS, which may contribute to the activation of ferroptotic cell death pathways. Moreover, the overexpression of Fpn, responsible for storing and regulating iron elements, playing a crucial role in the storage, release, and regulation of iron within cells (Jakaria et al., 2021), has been shown to partially improve memory impairment and reduce ferroptosis in mouse models of AD, providing further evidence for the crucial role of Fpn in the disease (Jakaria et al., 2021; Wang F. et al., 2022) (see Figure 2).

Furthermore, neuronal damage in AD can be attributed to glutamate excitotoxicity, which is caused by abnormalities in the function of the cystine/glutamate antiporter system (system Xc^−^) transporter. Increased oxidative stress, as indicated by elevated levels of oxidative damage, also exists in AD patients. Protein misfolding and abnormal metabolism of amino acids may further exacerbate iron accumulation, implicating ferroptosis as a potential mechanism in the progression of the disease (Jakaria et al., 2021). As one of the most important anti-oxidases, the conditional knockout of GPX4 in mice caused degeneration of hippocampal neurons, suggesting a possible link between ferroptosis and neurodegeneration in AD models (Park et al., 2021).

Although the precise role of ferroptosis in AD is still being investigated, the growing body of evidence strongly suggests its involvement in regulating the pathology of the disease, garnering significant attention from researchers in the field. Various strategies can potentially mitigate ferroptosis in AD, such as antioxidant therapy using compounds like vitamin E or Lip-1 to reduce neurodegeneration, iron chelation therapy to control iron levels and limit the generation of ROS, modulation of glutamate signaling pathways to address excitotoxicity, regulation of lipid metabolism to prevent LPO, and activation of GPX4 to safeguard cells against LPO and ferroptosis (Park et al., 2021). These approaches collectively offer promising avenues for intervening in the progression of AD and attenuating the neurodegenerative processes.

### 3.2 The role of ferroptosis in PD

PD is the second most prevalent chronic neurodegenerative disorder (Cannon and Greenamyre, 2013; Tufail and Hassan, 2020; Yang et al., 2017). It is distinguished by the formation of Lewy bodies and loss of dopaminergic neurons in the substantia nigra (SN) (Kosaka, 2014; Dickson, 2018; Han et al., 2023). The involvement of ferroptosisin PD has gained widespread recognition. PD patients exhibit anomalous iron buildup in the SN, accompanied by decreased GSH levels and increased lipid peroxides, which align with the biochemical characteristics of ferroptosis (Dong-Chen et al., 2023; Zeng et al., 2024; Yang et al., 2023). This suggests that ferroptosis contributes to the neurotoxicity observed in PD. Disruptions in iron homeostasis pathways are also implicated in PD. Recent data in the 6-Hydroxydopamine (6-OHDA) model of PD demonstrate that FTH1 links ferritinophagy and ferroptosis, offering a new perspective and potential target for pharmacological intervention (Tian et al., 2020). Alterations in iron regulatory proteins result in imbalances in iron storage and importation. Changes in ferritin levels, divalentmetal-ion transporter-1 (DMT1) expression, ceruloplasmin function, and ferroportin1 expression contribute to elevated cellular iron levels, potentially exacerbating the effects of ferroptosis on dopaminergic neurons.

**Figure 3:**
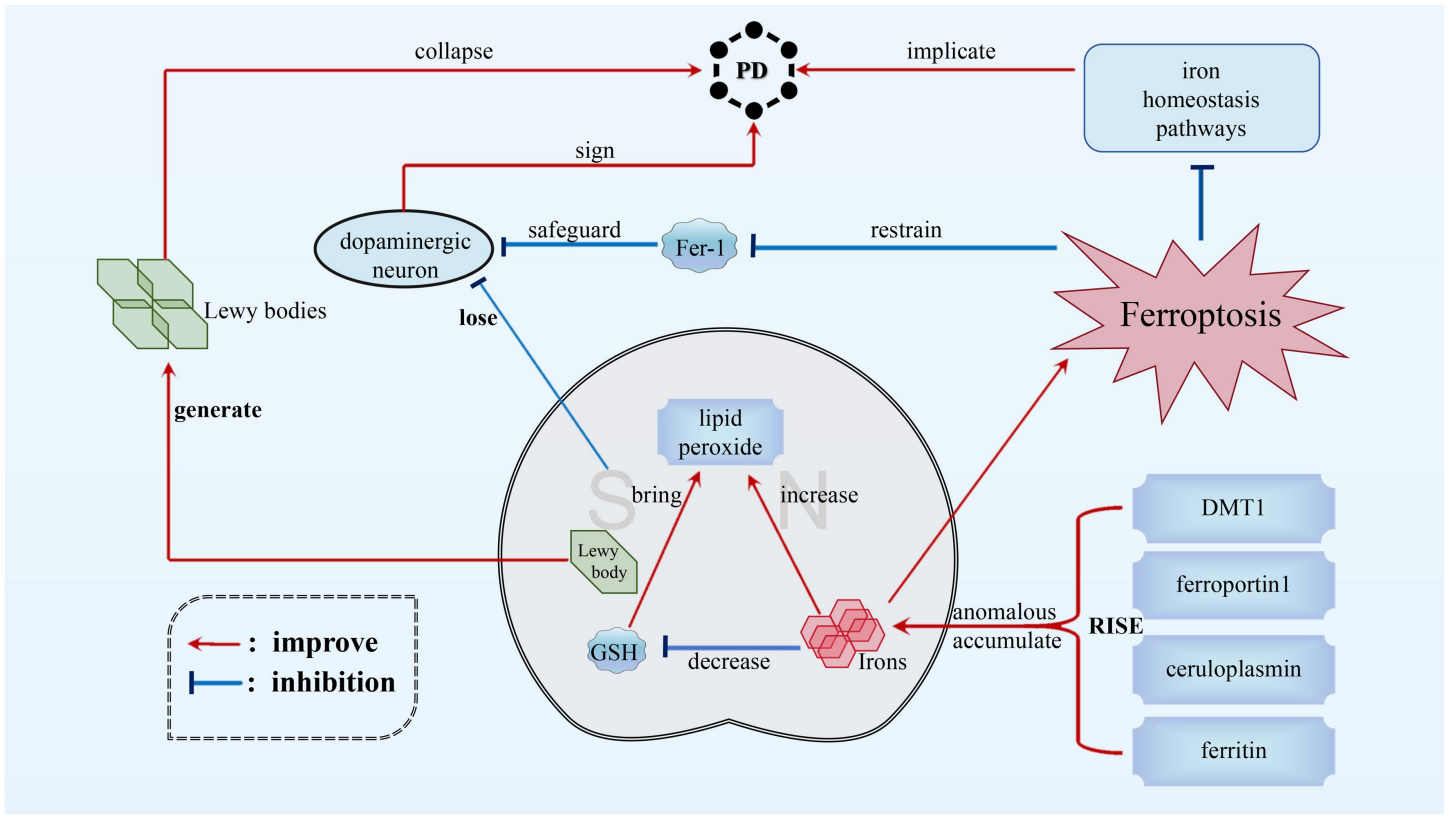
Ferroptosis in PD. The main pathological features of PD are the significant increase in Lewy bodies within the gray matter and the reduction in dopaminergic neurons. The destruction of dopaminergic neurons can be caused by neurotoxins produced by ferroptosis. This process disrupts iron homeostasis pathways and alters the function of ceruloplasmin. Furthermore, it increases the expression of ferroportin1 and DMT1, as well as ferritin levels. These changes result in abnormal iron accumulation in the substantia nigra (SN), ultimately leading to ferroptosis and creating a vicious circle. However, the compound Fer-1 has the ability to protect dopaminergic neurons and inhibit ferroptosis, thereby reducing the severity of PD.

Targeting ferroptosis pathways has been identified as a promising therapeutic strategy for PD. Inhibiting ferroptosis using compounds like Ferrostatin-1 (Fer-1) has demonstrated the ability to safeguard dopaminergic neurons against toxic substances and prevent neurodegeneration (Xia et al., 2024; Sun et al., 2020). Disparities in the expression of ferroptosis-related genes between normal individuals and PD patients, especially in the SN region, underscore the potential for developing novel treatments that regulate ferroptosis to address the mechanisms of dopaminergic neuron degeneration in PD (see Figure 3).

To potentially diminish the impact of ferroptosis in PD, a multifaceted approach can be considered. Strategies such as iron chelation therapy with agents like deferoxamine, the utilization of ferroptosis inhibitors like Fer-1, regulation of iron homeostasis through targeting iron regulatory proteins, bolstering antioxidant defenses to counteract oxidative stress, and gene therapy to modulate the expression of ferroptosis-related genes could collectively offer potential avenues to alleviate the impact of ferroptosis on dopaminergic neurons in PD. These measures aim to reduce abnormal iron accumulation, mitigate LPO, and enhance cellular resilience against ferroptosis. Furthermore, FTH1 links ferritinophagy and ferroptosis in the 6-OHDA model of PD (Tian et al., 2020; Li et al., 2021), which provides a new perspective for a pharmacological target in PD.

### 3.3 The role of ferroptosis in HD

**Figure 4:**
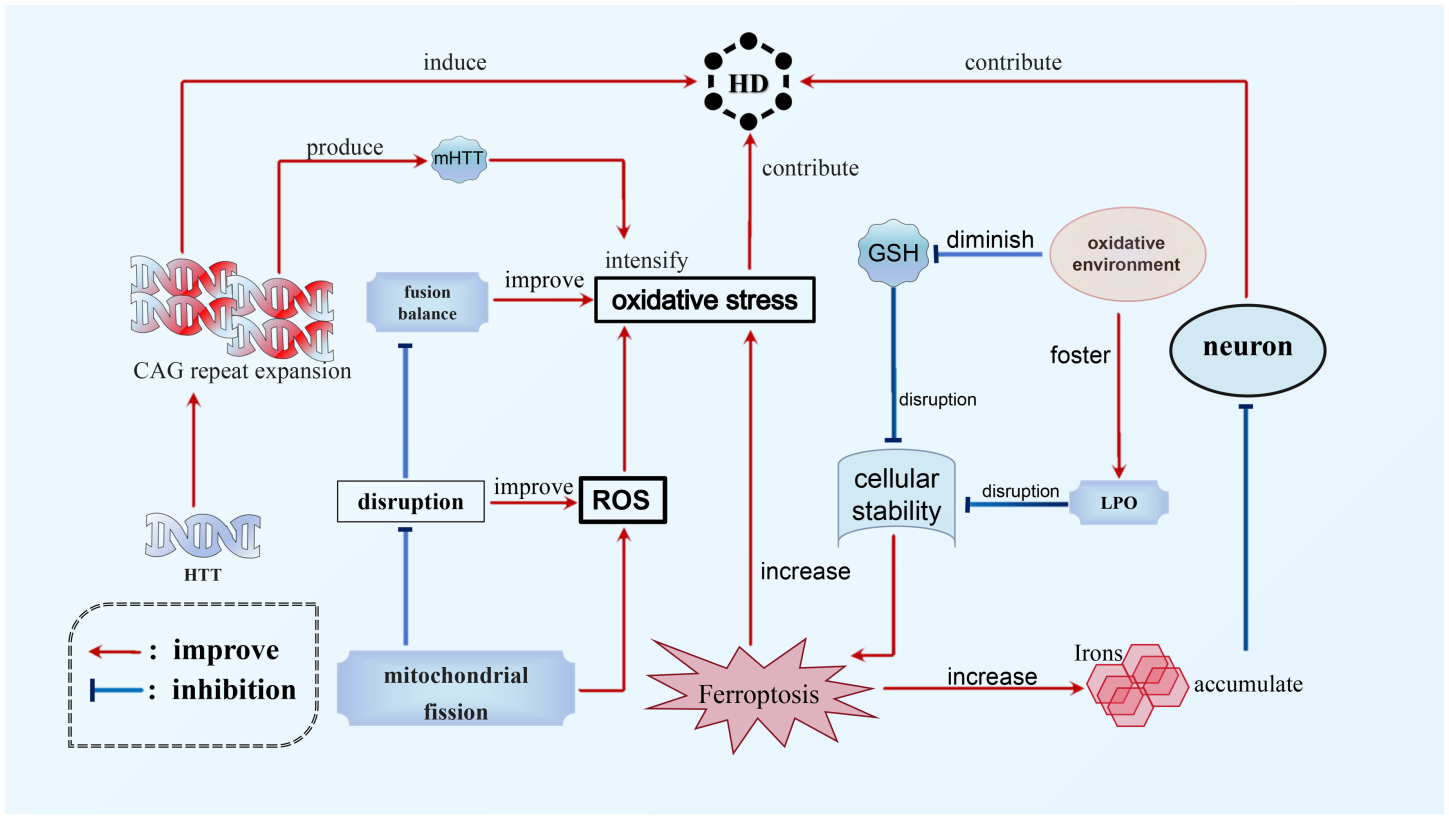
Ferroptosis in HD. The main cause of HD is the expansion of CAG repeats in HTT. This expansion not only directly leads to the occurrence of HD, but also contributes to the progression of the disease by worsening mHTT. Additionally, ferroptosis is another significant factor in the development of HD. There is a complex relationship between oxidative stress, ferroptosis, and HD. The accumulation of toxic iron during ferroptosis can promote the neurodegenerative process and worsen HD. Additionally, HD can increase levels of ROS, which, combined with mHTT, can exacerbate oxidative stress and further aggravate ferroptosis. This creates a vicious circle between these interconnected processes.

HD is a hereditary, lethal neurodegenerative disorder caused by abnormal Coronary angiography (CAG) repeats in the gene responsible for encoding the protein Huntingtin, which expresses ubiquitin (Tabrizi et al., 2020). The pathophysiology of HD involves the accumulation of toxic iron in neurons and an increase in oxidative stress, potentially contributing to the neurodegenerative process. Ferroptosis, which is a regulated form of cell death characterized by iron-dependent LPO, appears to play a significant role in the progression of HD. Experimental studies utilizing HD mouse models have demonstrated that inhibition of ferroptosis can prevent degeneration of spinal motor neurons and enhance motor function, thereby establishing a direct connection between the occurrence of ferroptosis and the advancement of HD (Chen et al., 2015). Moreover, the disruption of mitochondrial fission and fusion balance in HD leads to elevated levels of ROS and oxidative stress (Mi et al., 2019). The presence of mutant huntingtin protein (mHTT) further intensifies oxidative stress, thereby disrupting the delicate equilibrium of cellular stability (Wyttenbach et al., 2002). Subsequently, this oxidative environment fosters LPO while reducing glutathione levels, exacerbating the disruption of cellular homeostasis and potentially triggering the occurrence of ferroptosis. Empirical evidence indicates that individuals afflicted with HD demonstrate increased levels of LPO and diminished plasma GSH levels, thereby emphasizing the intricate association between oxidative stress, ferroptosis, and the pathophysiological mechanism of HD (Klepac et al., 2007; Yuan et al., 2017) (see Figure 4).

Multiple strategies present potential for mitigating ferroptosis in HD. These approaches encompass iron chelation for reducing iron levels, antioxidant therapy to counteract oxidative stress, activation of the Nfr2 pathway to fortify antioxidant defenses, modulation of mitochondrial function to diminish ROS production, and the development of medications that specifically target ferroptosis pathways, such as GPX4 inhibition. A combination of these strategies may hold the key to inhibiting ferroptosis in HD, offering promising avenues for managing the neurodegenerative processes associated with the disease and potentially decelerating its progression.

### 3.4 The role of ferroptosis in MS

**Figure 5:**
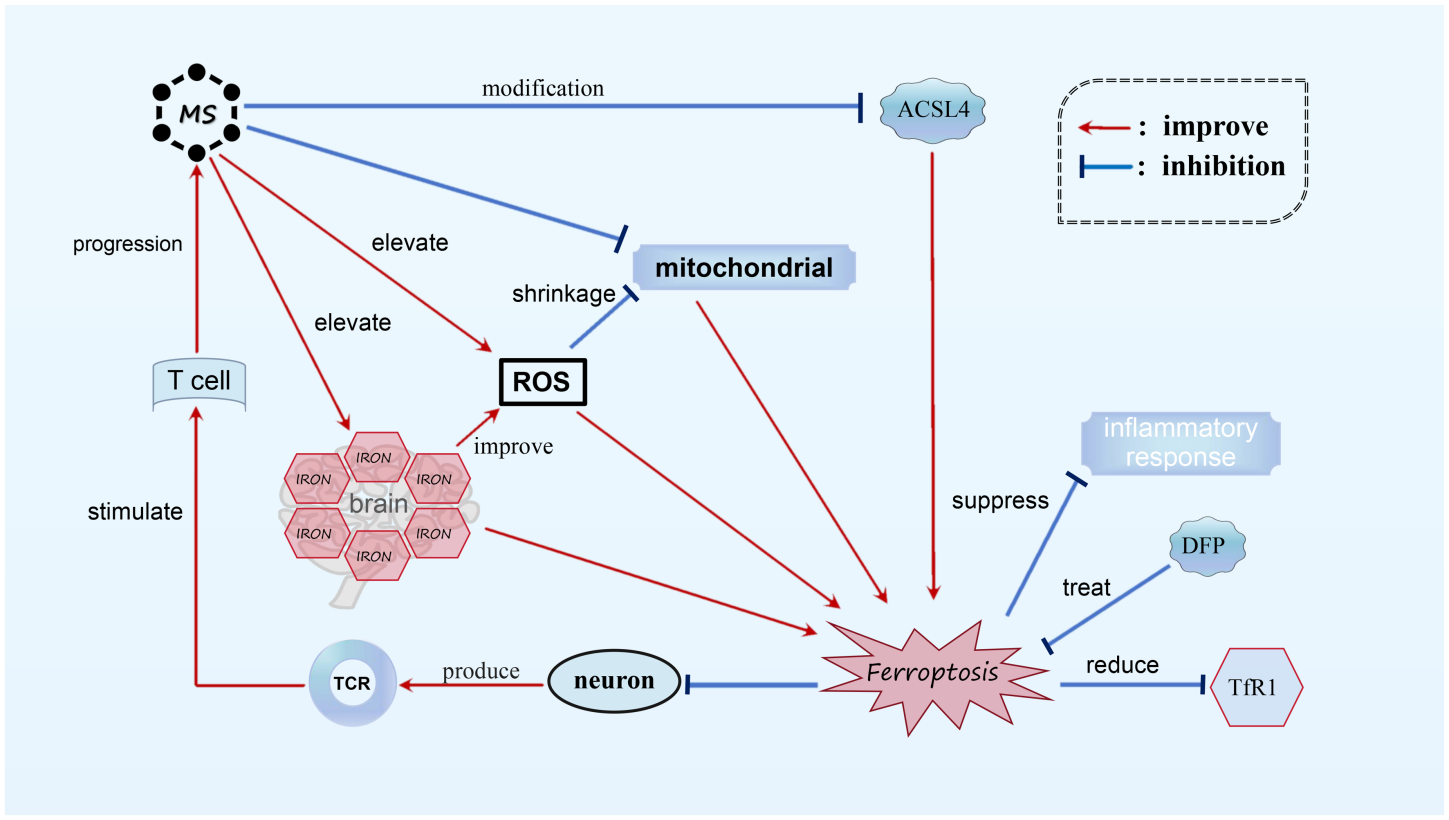
Ferroptosis in MS. In MS, we observed noticeable alterations in ACSL4, mitochondrial atrophy, and the accumulation of a substantial amount of ROS. Additionally, there is an abnormal increase in iron content within the brains of MS patients. These biochemical changes play a role in the occurrence of ferroptosis. Neurons undergoing ferroptosis can activate T cells through TCR stimulation, resulting in further deterioration of MS. It has been found that DFP is capable of reducing TfR1 content and suppressing the inflammatory response, thereby effectively treating MS by diminishing ferroptosis.

MS is an autoimmune inflammatory disorder that primarily affects the white matter of the CNS. The exact cause of the disease is still unknown (Reich et al., 2018), but it is characterized by neurodegeneration, inflammatory demyelination, primary demyelinating lesions, varying levels of axonal loss, and the proliferation of astrocytes and microglia (Reich et al., 2018; Kuhlmann et al., 2017). Through investigation, it has been discovered that the genetic composition responsible for a crucial protein involved in ferroptosis, known as acyl-CoA synthetase ACSL4, undergoes significant modifications in MS patients. These modifications correspond with ferroptosis, as observed in existing genomic databases of MS patients. In animal models of MS, such as the experimental autoimmune encephalomyelitis (EAE) mouse model, the accumulation of ROS and mitochondrial shrinkage have been observed. Magnetic resonance imaging (MRI) and histological analysis have also detected increased iron levels in both the white and gray matter of MS patients’ brains, with iron accumulation found in the white matter and macrophages of the basal ganglia (Dusek et al., 2016). These phenomena align with the characteristics of ferroptosis, indicating the presence of biochemical alterations related to ferroptosis in MS. (Luoqian et al., 2022) Furthermore, evidence from experiments suggests that neurons undergoing ferroptosis can stimulate T cell activation through the T cell receptor (TCR) signaling pathway in the EAE mouse model, worsening the progression of MS. Treatment with the iron-chelating agent Downstream Facing Port (DFP) in the EAE model has shown promising results, reducing Transferrin receptor protein 1 (TfR1) content, reversing iron overload, and inhibiting demyelination. By stabilizing inflammatory cells and suppressing the inflammatory response, DFP effectively inhibits ferroptosis in MS, making it a potential therapeutic option (Rayatpour et al., 2022) (see Figure 5).

Although the exact cause of MS remains unclear, it is apparent that iron death plays a role in MS, with ferroptosis potentially contributing to its development. More research is needed to understand the genetic and chemical mechanisms involved in MS. Analyzing experimental data has shown a potential relationship between ferroptosis and MS, with both factors influencing each other. In future studies, exploring the inhibition of ferroptosis could slow down the progression of MS, while reducing ferroptosis-related characteristics in affected areas could further validate the impact of ferroptosis on MS.

### 3.5 The role of ferroptosis in ALS

ALS, also referred to as motor neuron disease (MND), is a progressive neurodegenerative disorder that impacts both upper and lower motor neurons, resulting in muscle weakness and ultimately respiratory failure and mortality within a few years of symptom onset (Wang T. et al., 2022). Anomalous accumulation of iron has been observed in the spinal cord and cerebral regions of individuals with ALS and animal models (Devos et al., 2020). This dysregulation of iron is associated with heightened LPO and oxidative stress, contributing to neuronal damage. Research has demonstrated increased levels of markers of ferroptosis, such as 4-HNE, in individuals with ALS. The suppression of crucial pathways involved in safeguarding cells against ferroptosis, such as the GSH/GPX4 axis, has been associated with degeneration of motor neurons and progression of the disease in ALS. Moreover, the FSP1-CoQH2 system and the GTP cyclohydrolase I-BH4 (GCH1-BH4) system have been identified as potential targets in ALS (Wang et al., 2023a,b; Wang T. et al., 2022). Modulating these systems could potentially offer therapeutic benefits by mitigating ferroptosis and oxidative stress in individuals with ALS.

Few studies have been conducted on ALS and ferroptosis, and the precise relationship between them remains unclear. Further exploration is necessary to understand this connection in more detail.

## 4 Conclusions and perspectives

In this review, we present a concise overview of the current knowledge regarding the characterization, induction, and inhibition of ferroptosis (specifically focusing on iron death inhibitors), as well as its role in regulating the occurrence and progression of multiple neurodegenerative diseases, namely AD, PD, HD, MS and ALS. The current study indicates that ferroptosis plays a pivotal role in neurodegenerative diseases and has significant potential as a target for therapeutic interventions. However, the exploration of ferroptosis still faces formidable challenges. Firstly, the investigation of ferroptosis in cognitive dysfunction-related diseases is still in its nascent stage, and its underlying molecular mechanisms remain elusive. Hence, the identification of specific markers of ferroptosis is crucial to comprehensively and extensively study its intricate process. Secondly, the majority of the available data is derived from experimental studies, necessitating the inclusion of more clinical approaches. For instance, experimental employment of iron chelators and antioxidants has proven an effective means to disrupt the ferroptotic process in neurodegenerative diseases. Nonetheless, their efficacy is reduced when tested in human subjects, highlighting the need to explore signaling molecules implicated in alternative pathways triggering iron accumulation. Furthermore, ferroptosis serves not only as a detrimental process but also as a crucial physiological defense mechanism. By inducing cell death in cancer cells, ferroptosis effectively hampers tumour progression and metastasis (Stockwell, 2022; Jiang et al., 2021; Tang D. et al., 2021). In other words, ferroptosis inhibitors used to treat neurodegenerative diseases may facilitate the growth of tumours, such as butylated hydroxytoluene (Faraji et al., 2024). Consequently, prior to endorsing the clinical utilization of ferroptosis inhibitors in treating neurodegenerative diseases or other ailments, more comprehensive mechanistic investigations regarding the potential adverse effects of this compound are imperative. Additionally, the relationship between ferroptosis and other forms of cell death mechanisms remains inadequately understood, and there is a dearth of research on the combination of drugs. Therefore, future efforts should focus on elucidating the intricate associations and mechanisms linking ferroptosis to other well-known cell death pathways, as well as conducting experiments involving the concurrent administration of various agents. These endeavors hold the potential to illuminate innovative therapeutic interventions and strategies.
